# Institutional Notes and News

**Published:** 1910-03-26

**Authors:** 


### Institutional Notes and News.

**Samaritan Free Hospital for Women, Marylebone Road, W.**

The annual meeting of Governors was held in the Board Room on Tuesday, March 8. The Chairman, Lieut.-Col. J. T. Woolrych Perowne, in presenting the report for 1909, remarked that the hospital had had a very successful year in every respect. No fewer than 904 patients were treated in the in-patient department, a record number; and considering the very serious nature of the cases received, it was evident that the hospital, with fifty-six available beds, had been worked to its fullest capacity. The out-patient department had benefited nearly 4,000 poor women. Financially, too, the year had been a good one—general fund income being £5,574 and the expenditure £5,575. The debt of £1,200 on the building fund had been reduced by £1,050 during 1909, and the remaining £150 had been paid off this year partly from the general fund, so that the building-fund account was now closed. The Samaritan Fund, with an income of about £100, helped no fewer than 295 of the poorest patients. Contributions, particularly annual subscriptions, are much needed to meet current expenses, and will be gratefully received by Messrs. Scott's branch of Parr's Bank, 1 Cavendish Square, W., or by the Secretary, Mr. W. Guntrip King, at the hospital.

**University College Hospital.**

It is satisfactory to hear that subscriptions are coming in to meet the special appeal for £10,000 which University College Hospital intends to raise by June of this year. Readers of The Hospital will hardly need reminding that while the splendid gift of £200,000 from Sir John Blundell Maple enabled the hospital to be rebuilt, it did not provide for the annual maintenance. The very efficiency of the new model added £10,000 a year to the expenses of upkeep, and last year University College Hospital suffered more perhaps than other institutions from the scarcity of legacies and charitable gifts of all sorts. It is worthy of record, therefore, that the following gentlemen have already come forward: Mr. W. C. Alexander and Mr. Roger Beck, £100 each; Mr. T. J. Davies, £21; Mr. E. Raphael, £20; B.M., £20; Mr. Cecil Raphael, £10 10s., and these, we are glad to say, are only a few among the supporters of the appeal. Nevertheless the annual report records that 1910 began with a total deficit of £8,000. Last year the in-patients numbered 3,369, and 52,709 persons were treated in the out-patient department.

**Lord Rosebery and Ventnor Hospital for Consumption.**

Lord Rosebery presided at the annual meeting of the National Hospital for Consumption at Ventnor, which was held at the London offices in Buckingham Street. He said that for a charity report, in a year highly unpropitious for charitable contributions, the hospital's budget for the previous year was one that might be regarded with considerable satisfaction. The expenditure was less by £227, and the cost per occupied bed per week had been reduced. The accounts read as follows: Receipts, £20,287, including £7,545 from legacies; disbursements, in round figures, £13,000; and of the remainder, £6,000 had been invested, and the deficit of £584 with which the year had started had been paid off. Thus the hospital was left in the happy position of carrying forward a balance of £568, which was a triumphant reversal of last year's opening procedure. The beds have been increased from 150 to 163, and the average length of stay has gone up to eighty-nine days. Lord Rosebery added the important fact that, according to the Local Government Board's returns, the death-rate from tuberculosis has been halved since the hospital was founded. In 1867 it was 24.4 per 10,000; it is 12.1 per 10,000 to-day. Lord Rosebery, amid laughter, then reiterated his "timid complaint" of last year that no grant was received by the hospital from the King's Fund. He reiterated also his fear that this complaint again would be unavailing.

**London Temperance Hospital.**

When this hospital was founded, thirty-six years ago, there was a proviso that in any case regarded as necessary the medical staff should be free to administer alcohol. We learn from the latest annual report that such necessary cases, each of which has been placed on record, have only arisen eighty-five times. The in-patients for the same period have numbered almost 30,000, 1,279 of whom were treated last year, while 27,566 cases were treated in the out-patient department. It is satisfactory to observe that the accounts have a surplus to their credit, the figures reading income £12,105, and expenditure £11,141. A second meeting was held on the same day to enforce the results of the hospital's thirty-six years of experience on the attention of the profession and the public. Sir J. H. Roberts, M.P., spoke, summing up these results by the remark that the old belief that alcohol was essential in medicine had been now effectually destroyed. Sir T. Vesey Strong, chairman of the Board of Management, presided.

**Mount Vernon Hospital.**

The fiftieth annual report of the Mount Vernon Hospital for Consumption and Diseases of the Chest, Hampstead and Northwood, was presented at the recent annual meeting, when Captain Jessel, M.P., presided. At Hampstead the daily average number of occupied beds was 221, and last year the attendances in the out-patient department totalled 14,558. It is only lack of funds which prevents the accommodation at Northwood from being greatly increased. Last year the accounts showed a satisfactory balance, the ordinary income being £18,884, while the expenditure was £17,117. The institution is thus in a position to be especially proud of its jubilee year's work.

**Royal Sea Bathing Hospital, Margate.**

Lord Biddulph presided over the annual meeting of the Governors of the Royal Sea Bathing Hospital, Margate, which was held at the London offices. He pointed out the need for free beds, additional wards, better day rooms for the men patients, a waiting room for out-patients, and a dentistry. The ordinary income was £9,651, and the expenditure £9,577, which mark a financial improvement, but this, we understand, has been gained by modifying the principle of free admission; were that principle to be completely applied £4,000 more would be necessary to balance the accounts. Lord Biddulph further remarked that better results would be recorded if patients applied for treatment at the first stages of the disease. It is hoped that the Prince and Princess of Wales may honour the hospital with a visit before very long.

**The Poplar Hospital for Accidents.**

Mr. J. G. Broodbank, acting-chairman of the hospital, presided at the recent annual meeting, and remarked that it was very unfortunate that Mr. Sydney Holland had severed his active connection with the hospital, though it had the advantage of keeping him as nominal chairman. The hospital could be congratulated on still keeping out of debt, and there was good reason for believing that the £500 a year from King

Edward's Fund would become a permanent grant. With one exception, the number of patients treated during the year was the largest on record, and if the kind of case were taken into account, the past year had been the most important in the hospital's history. The ordinary expenditure showed a saving of £2,100, due entirely to the fact that so much money had not been spent on painting and cleaning as usual, but this saving could be only temporary, because some of the wards had not been thoroughly cleaned for fourteen years. The convalescent home proved its usefulness every month, and he could not be too thankful that they had embarked on that enterprise. The hospital felt especially grateful to Sir Hudson Kearley for having presided at the annual dinner, for his presidency there had identified the Port Authority with the hospital, which was important for both. The report showed that considerable correspondence had taken place between the London County Council and the hospital with regard to the treatment of some of the children. The management was very sorry that it could not come into arrangement with the Council; but it was not the fault of either of them. There was not so much objection in attending to children's teeth as to skin and other diseases, and the matter would be brought before the management soon. The report again gratefully recognised the good work done by Miss Bland, the matron, who, though she had been there fourteen or fifteen years, still worked as well as ever. At the instance of the Chairman, a special resolution of appreciation and thanks was accorded to the Secretary, Mr. Percy Rogers, who wished it to include his assistant, Mr. Loates. The actual figures of the report were: in-patients 1,457, out-patients and casualties 53,086, and ordinary expenditure was £9,049.

**Infants' Hospital.**

Mr. J. S. Fletcher, M.P., presided over the annual governors' court of the Infants' Hospital, Vincent Square, Westminster. The number of patients admitted was 415, and a new departure was made by the decision, come to at the end of last year, to set up an out-patient department. Here patients are seen every day at 2 P.M. "Since November last," says the report, "all the milk used in the hospital has been received from a farm at Combe Bank, Sevenoaks," which is directly under the control of the hospital's staff. Special refrigerating machinery in connection with the milk laboratory has also been established. The scheme for receiving paying probationers at the hospital has been made use of by many ladies anxious for experience in infant nurture. We are glad to see that the Infants' Hospital has taken to heart the advice tendered in The Hospital recently, and produced a report which is quite charmingly printed and bound.

**Brompton Hospital for Consumption.**

The daily average number of occupied beds last year at the Brompton Hospital for Consumption was 457, a number, said Lord Cheylesmore, chairman of the committee of management, equal to about half the total number of free beds existing for consumption patients in the country. The hospital and sanatorium at Frimley had admitted 1,643 in-patients, the average stay of whom was 101 days. In the out-patient department 50,000 attendances were recorded. It should be added further that the scheme which the Brompton Hospital has inaugurated for voluntary notification led to 747 cases being notified to the medical officers of health. Subscriptions, other than annual contributions, again showed a most unfortunate diminution, in spite of the fact that more than a dozen cots have been added so that younger children can be better received. Special mention was made of the great loss which the hospital has suffered from the death of Mr. T. P. Beckwith, who for 30 years had been chairman of the committee of management. The annual meeting of governors was presided over by the Duke of Wellington.

